# Elevated CO_2_ Alters the Physiological and Transcriptome Responses of *Pinus densiflora* to Long-Term CO_2_ Exposure

**DOI:** 10.3390/plants11243530

**Published:** 2022-12-15

**Authors:** Tae-Lim Kim, Hyemin Lim, Hoyong Chung, Karpagam Veerappan, Changyoung Oh

**Affiliations:** 1 Department of Forest Bioresources, National Institute of Forest Science, Suwon 16631, Republic of Korea; 2 3BIGS CO. Ltd., Hwaseong 18469, Republic of Korea

**Keywords:** carbon dioxide, climate change, open-top chamber, physiological response, transcriptome, *Pinus densiflora*

## Abstract

Physiological response and transcriptome changes were observed to investigate the effects on the growth, metabolism and genetic changes of *Pinus densiflora* grown for a long time in an environment with an elevated atmospheric CO_2_ concentration. Pine trees were grown at ambient (400 ppm) and elevated (560 ppm and 720 ppm) CO_2_ concentrations for 10 years in open-top chambers. The content of nonstructural carbohydrates was significantly increased in elevated CO_2_. It was notable that the contents of chlorophylls significantly decreased at an elevated CO_2_. The activities of antioxidants were significantly increased at an elevated CO_2_ concentration of 720 ppm. We analyzed the differences in the transcriptomes of *Pinus densiflora* at ambient and elevated CO_2_ concentrations and elucidated the functions of the differentially expressed genes (DEGs). RNA-Seq analysis identified 2415 and 4462 DEGs between an ambient and elevated CO_2_ concentrations of 560 ppm and 720 ppm, respectively. Genes related to glycolysis/gluconeogenesis and starch/sucrose metabolism were unchanged or decreased at an elevated CO_2_ concentration of 560 ppm and tended to increase at an elevated CO_2_ concentration of 720 ppm. It was confirmed that the expression levels of genes related to photosynthesis and antioxidants were increased at an elevated CO_2_ concentration of 720 ppm.

## 1. Introduction

The rapid increase in the concentration of CO_2_ in the atmosphere since the beginning of the industrialization era has caused global problems, such as global warming and desertification. The concentration of CO_2_ in the atmosphere has increased to 400 ppm from 280 ppm before industrialization and is expected to reach 550–700 ppm in 2050 and 650–1200 ppm in 2100 [1]. In addition, global warming is expected to become more severe. Carbon assimilation accompanying photosynthesis in forests, especially trees, has the effect of reducing CO_2_ in the atmosphere as carbon sequestration [2]. Therefore, the conservation and expansion of forests are important issues worldwide. Researchers are preparing to respond appropriately to the changing atmospheric environment by examining the genetic, physiological and morphological responses of trees under high-concentration CO_2_ conditions. It has been reported that conditions with high CO_2_ concentrations particularly affect the physiological response of seedlings and significantly affect carbon assimilation, water use efficiency and metabolism [3,4,5,6]. These conditions are also known to affect tree height, diameter, number of leaves, size, number of branches, growth rate and biomass [7]. The concentration of CO_2_ in the atmosphere strongly influences photosynthesis throughout the life of plants and functions as selective pressure for environmental adaptation. CO_2_ is an essential component of photosynthesis and affects the efficiency of carbohydrate storage, which leads to changes in the biomass and net production of plants [8]. Due to long-term processing and observation difficulties, the investigation of responses and evidence in adult trees is insufficient. Several studies have focused on understanding the effect of elevated CO_2_ on crops and plants using open-top chambers (OTCs) and free-air CO_2_ enrichment (FACE) [8,9,10,11]. Runion et al. [12] reported the positive effects of elevated CO_2_ on conifer (longleaf pine) biomass and carbon accumulation in a model regenerating a longleaf pine community composed of five species.

Pine is a representative coniferous evergreen tree widely distributed in Korea, Japan and China, and there are approximately one hundred species worldwide [13]. *Pinus densiflora* (*P. densiflora*) is a pine tree inhabiting the Korean mountains that has long been used as wood for construction, furniture and firewood. Even today, it is an important tree species economically, ecologically and culturally to the extent that afforestation has been promoted at the government level for this purpose [14]. Despite their importance, their habitats are declining due to climate change and global warming [15]. Therefore, we studied the effect of long-term growth of pine trees in an atmospheric environment with increased CO_2_, which is an important factor in atmospheric environmental change and has a great effect on plant growth. Pine seedlings were grown for 10 years in open-top chambers (OTCs) while maintaining three different CO_2_ concentrations, *viz.,* 400 ppm (OTC1), 560 ppm (OTC2) and 720 ppm (OTC3).

Research on the response of plants to high concentrations of CO_2_ is steadily progressing because a rapid increase in CO_2_ is expected in the next few decades. In particular, it is difficult to observe the responses of trees exposed to high concentrations of CO_2_ for a long time, but it will be a preparation for important environmental changes. We analyzed the physiological and transcriptional differences found in pine leaves grown at different concentrations of CO_2_ for 10 years. RNA-seq analysis has been useful to identify physiological and molecular reaction mechanisms for biotic and abiotic stresses in pine trees [11,16,17,18]. However, there has been no report on transcriptome analysis of pine trees that have grown while exposed to high concentrations of CO_2_ for 10 years. Therefore, we aimed to study the physiological and transcriptional changes in pine trees grown under ambient and elevated CO_2_ concentrations and to comprehensively correlate physiological parameters with changes in transcript abundance.

## 2. Results

### 2.1. Growth and Physiological Changes in Response to Elevated CO_2_

**Figure 1:**
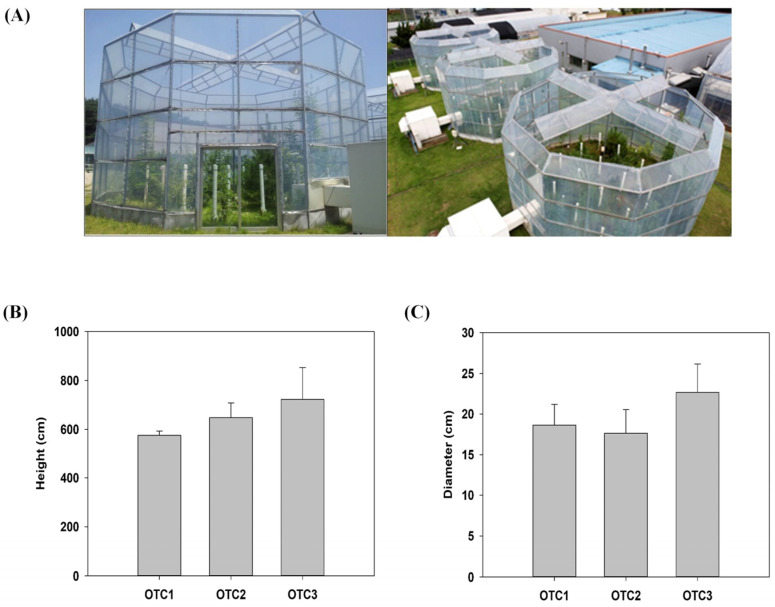
Changes in the phenotypes of *P. densiflora* as affected by elevated CO_2_ concentrations after 10 years of treatment. (**A**) A photograph of the whole open-top chamber (OTC) facility. The effects of elevated CO_2_ on the shoot growth (**B**) and diameter (**C**) of the pine trees. The values are the means ± SDs (*n* = 3).

To investigate the high-CO_2_ effect during 2010–2019, the diameter, height, antioxidants, carbohydrates and photosynthetic pigments of pine trees under differential CO_2_ treatment were measured. We observed the phenotypes of shoots and measured the physiological changes in the pines’ leaves. The height and diameter of the pines grown with elevated CO_2_ concentrations of 560 ppm and 720 ppm tended to increase (Figure 1). However, there were no statistically significant differences observed between ambient and elevated CO_2_ pine trees. The leaf chlorophyll content was correlated with photosynthetic activity, and the chlorophyll levels were affected by elevated CO_2_. The chlorophyll a content of pine trees was significantly reduced at an elevated CO_2_ concentration of 720 ppm (Table 1). Furthermore, reduced chlorophyll b content was also observed at elevated CO_2_ concentrations of 560 ppm and 720 ppm compared to the controls (Table 1). In addition, the total chlorophyll content of pine was also significantly reduced at an elevated CO_2_ concentration of 720 ppm. There was an increasing ratio of chlorophyll a/b at elevated CO_2_ concentrations of 560 ppm and 720 ppm. In the case of carotenoids, there was an observed change in carotenoids at an elevated CO_2_ concentration of 560 ppm.

To estimate the effect of elevated CO_2_ concentrations on the carbon metabolite contents of pine, glucose, fructose, sucrose, total soluble sugar and starch were investigated (Table 2). The pine trees grown at higher concentrations of CO_2_ tended to have higher glucose, fructose and sucrose contents than those grown at ambient concentrations. The glucose content at an elevated CO_2_ concentration of 720 ppm and the sucrose content at an elevated CO_2_ concentration of 560 ppm were significantly higher than those under ambient conditions. The starch concentration in pine leaves was increased at an elevated CO_2_ concentration of 560 ppm. Except for the above findings, fructose and others seemed to increase slightly, but there were no statistically significant differences. Both elevated CO_2_ conditions increased the concentrations of total soluble sugars in the leaves of pine.

**Figure 2:**
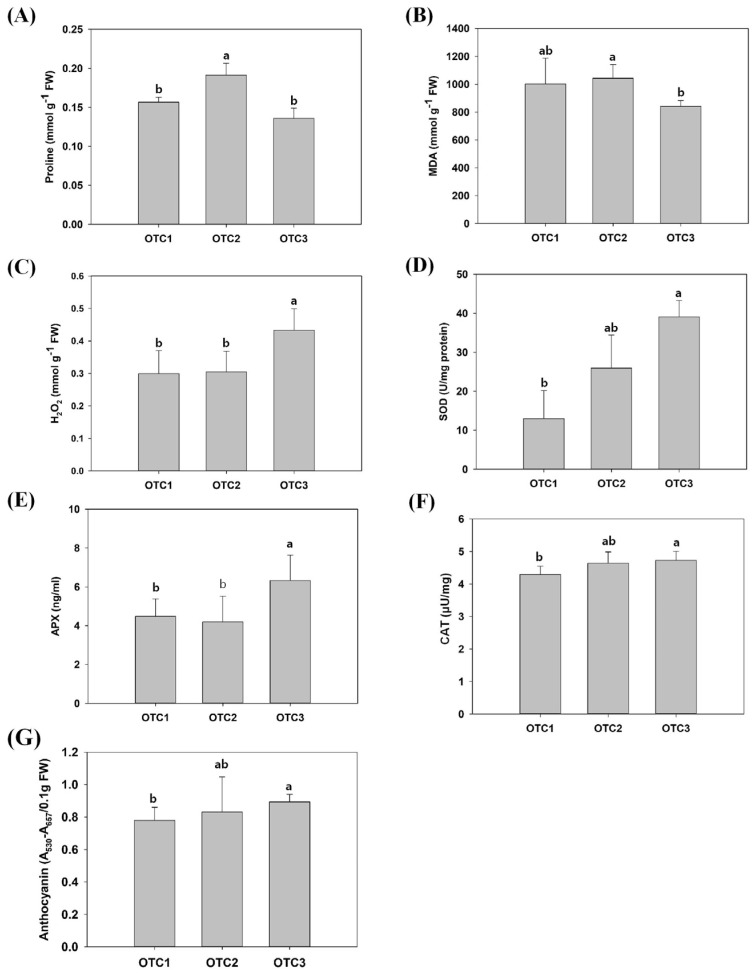
Effects of CO_2_ treatment on the metabolites and carbohydrate contents. (**A**) Malondialdehyde (MDA). (**B**) Proline. (**C**) Hydrogen peroxide (H_2_O_2_). (**D**) Superoxide dismutase (SOD). (**E**) Ascorbate peroxidase (APX). (**F**) Catalase (CAT). (**G**) Anthocyanin. Different lowercase letters indicate significant differences (ANOVA with Tukey’s HSD, *p* < 0.05).

To investigate the effects of elevated CO_2_ on the stress-responsive elements, malondialdehyde (MDA), H_2_O_2_, superoxide dismutase (SOD), catalase (CAT), ascorbate peroxidase (APX), anthocyanin and proline contents were measured (Figure 2). Proline concentration, a representative indicator of drought stress, increased only at a CO_2_ concentration of 560 ppm [19]. In addition, the levels of MDA showed a significant difference only between elevated CO_2_ concentrations of 560 ppm and 720 ppm. The level of MDA in plant tissue is commonly used as an index of oxidative lipid injury and tissue damage induced by environmental stress [20]. The H_2_O_2_ content was measured to determine the state of the ability to remove free radicals under oxidation stress. SODs play important roles in antioxidant defense and redox regulation [21]. Here, an elevated CO_2_ concentration of 720 ppm significantly increased the concentration of H_2_O_2_ in the leaves of pine. Additionally, at an elevated CO_2_ concentration of 720 ppm, the activities of antioxidant-related enzymes such as SOD, CAT, APX and anthocyanins were significantly increased.

### 2.2. RNA Sequencing and Assembly

The transcriptome of *P. densiflora* was sequenced and de novo assembled, and the assembly statistics are shown in Table 3. The data quality was assessed with the Illumina assessment score, and the result showed a good quality score (Q30) of 95% in all sequenced samples. The raw reads were quality trimmed to remove adapters and redundant and short reads. Approximately 29,409,471 filtered reads at the ambient CO_2_ concentration of 400 ppm (OTC1), 30,821,126 reads at the elevated CO_2_ concentration of 560 ppm (OTC2) and 28,728,623 reads at the elevated CO_2_ concentration of 720 ppm (OTC3) were generated (Table 3). The clean reads were de novo assembled using Trinity (Table 3). A total of 70.72% (OTC1), 70.31% (OTC2) and 68.72% (OTC3) of the clean reads were mapped with assembled reference sequences.

**Figure 3:**
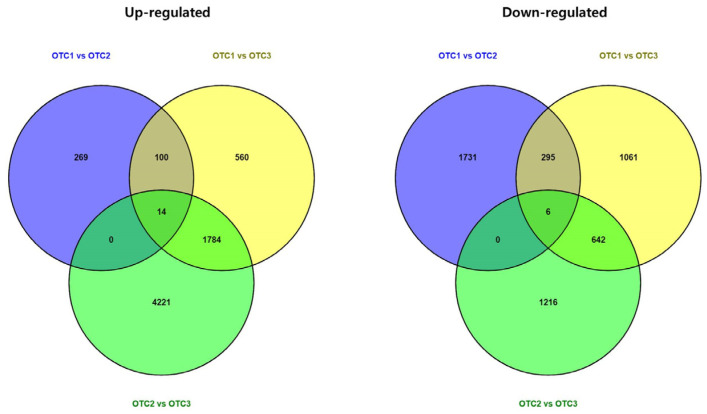
Overview of differential gene expression (DEG) in all comparison groups. Each species was treated with three different concentrations of CO_2_. OTC1 (control, ambient CO_2_ concentration of 400 ppm), OTC2 (elevated CO_2_ concentration of 560 ppm) and OTC3 (elevated CO_2_ concentration of 720 ppm).

We intended to identify the transcriptional adaptation of pine trees to increased CO_2_ concentrations at the gene level. To this end, differentially expressed gene analysis was performed on the transcriptome of pine trees grown under different concentrations of CO_2_. In total, 2415, 4462 and 7883 genes were differentially regulated in the OTC1 vs. OTC2, OTC1 vs. OTC3 and OTC2 vs. OTC3 comparisons, respectively (Figure 3).

### 2.3. Functional Annotation and Gene Set Enrichment Analysis

The biological functions of the DEGs were annotated using Gene Ontology (GO), and similar patterns of enrichment were observed in all the comparisons studied. Accordingly, 50, 345 and 623 upregulated genes and 221, 262 and 196 downregulated genes were GO enriched in the OTC1 vs. OTC2, OTC1 vs. OTC3 and OTC2 vs. OTC3 comparisons, respectively. The DEGs were mainly categorized into ‘biological process’, ‘cellular component’ and ‘molecular function’ based on the GO terms. Under the biological process categories, the greater number of DEGs fell under ‘metabolic process’, ‘responsive to stimulus’, ‘localization’ and ‘biological regulation’. ‘Membrane’, ‘cytosol’, ‘protein containing complex’ and ‘nucleus’ GO terms were the most abundant in the cellular components. Of the molecular functions, most of the genes were categorized as ‘ion binding’, ‘nucleotide binding’, ‘nucleic acid binding’, ‘hydrolase activity’ and ‘transferase activity’.

**Figure 4:**
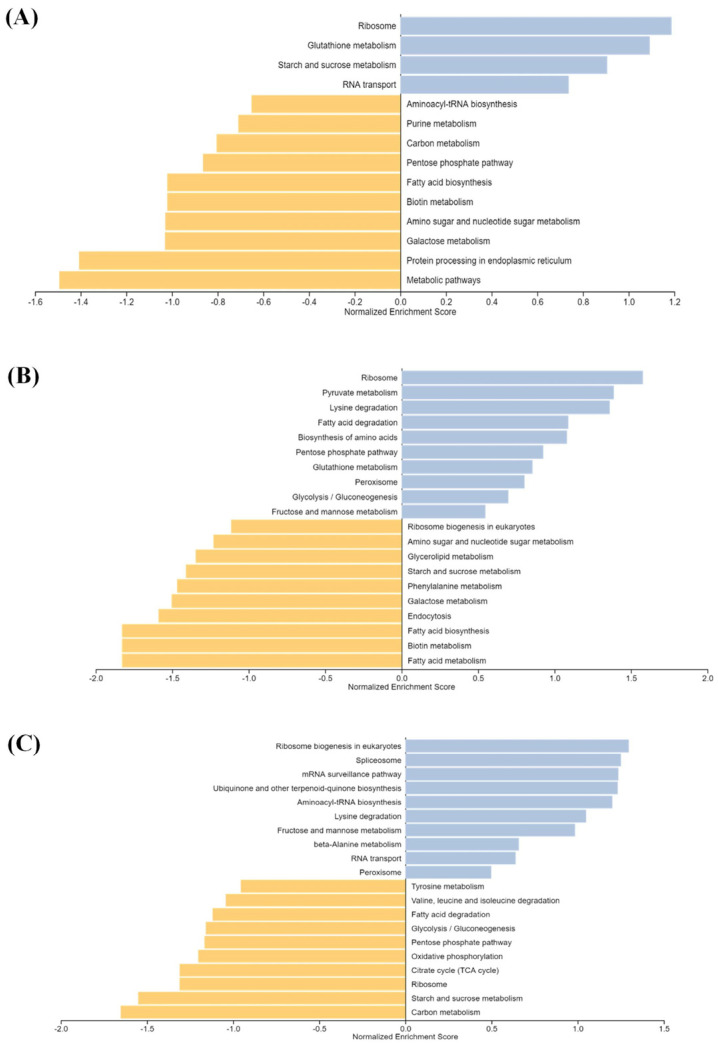
KEGG pathway enrichment analysis for (**A**) OTC1 vs. OTC2, (**B**) OTC1 vs. OTC3, (**C**) OTC2 vs. OTC3. Each species was treated with three different concentrations of CO_2_. OTC1 (control, ambient CO_2_ concentration of 400 ppm), OTC2 (elevated CO_2_ concentration of 560 ppm) and OTC3 (elevated CO_2_ concentration of 720 ppm).

KEGG enrichment analysis of DEGs of the respective groups was performed to identify the pathways most enriched, and the top 10 pathways were listed using WebGestalt [22]. Significantly, ‘ribosome’, ‘glycolysis/gluconeogenesis’, ‘biosynthesis of amino acid’, ‘tropane, piperidine and pyridine alkaloid biosynthesis’, ‘carbon metabolism’, ‘glutathione metabolism’ and ‘pentose phosphate pathway’ were the most observed common pathways (Figure 4). The majority of DEGs clustered into GO terms related to growth (ribosome) and carbon metabolism. The transcripts belonging to ribosomal proteins, RNA transport proteins and synthesis of amino acids were upregulated in the elevated CO_2_ concentrations compared to ambient CO_2_-treated trees, which might indicate an increase in growth induced by elevated CO_2_ [23]. Notably, the top enriched pathways included overall carbon metabolism; specifically, certain genes involved in oxidative phosphorylation, the pentose phosphate pathway and the TCA cycle were upregulated in the elevated CO_2_ group. The transcripts encoding enzymes involved in starch and sucrose metabolism, glycolysis/gluconeogenesis and sugar pathways were also upregulated under increased CO_2_ concentrations, together depicting a cycle of energy synthesis and utilization of pine’s increased photosynthesis as a result of elevated CO_2_ treatment, consistent with a previous study by Wu et al., 2019 [11]. Overall, the *P. densiflora* transcriptome analysis under elevated CO_2_ revealed upregulated photosynthesis and carbon metabolism.

### 2.4. Photosynthesis and Carbon Metabolism

**Figure 5:**
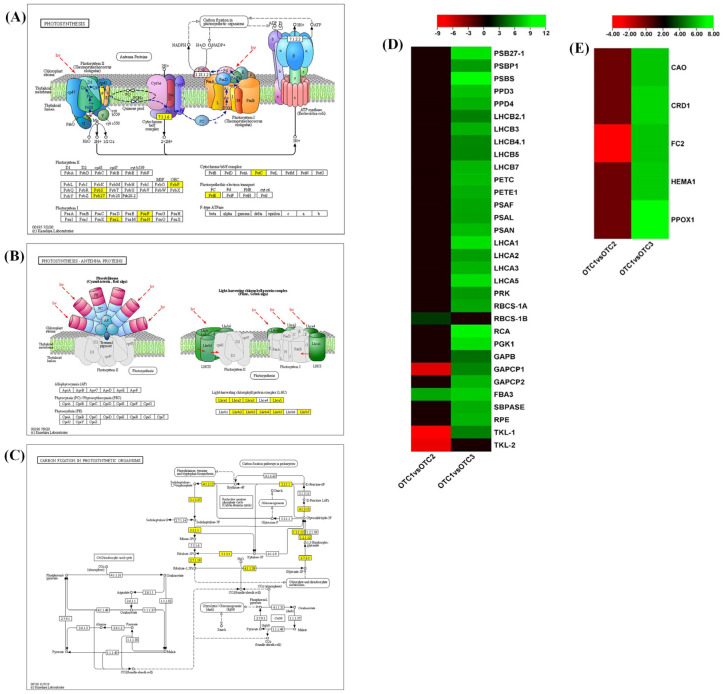
KEGG pathway analysis of photosynthesis. KEGG pathway illustration showing DEGs involved in (**A**) photosynthesis, (**B**) photosynthesis antenna proteins and (**C**) carbon fixation in photosynthetic organisms. The yellow boxes indicate the DEGs in response to increased CO_2_. (**D**) Heatmap showing expression changes in the photosynthetic pathway. (**E**) Heatmap showing expression changes in chlorophyll synthesis and cycle pathway. The color bar shows the log2fold changes scale. Each species was treated with three different concentrations of CO_2_. OTC1 (control, ambient CO_2_ concentration of 400 ppm), OTC2 (elevated CO_2_ concentration of 560 ppm) and OTC3 (elevated CO_2_ concentration of 720 ppm).

Photosynthesis is a biological process limited by the availability of nutrients such as carbon (CO_2_), nitrogen, water and radiation. Increasing temperature and atmospheric CO_2_ will impact the overall rate of photosynthesis and energy metabolism. In this study, increasing the CO_2_ concentration resulted in the increased expression of photosynthetic genes. Overall, the expression levels of 19 photosystem-related genes and 14 genes related to the Calvin cycle and pentose phosphate pathway (PPP) were differentially regulated (Figure 5A–D). These genes were marked into ‘photosynthesis’, ‘photosynthesis-antenna proteins’ and ‘carbon fixation of photosynthetic organism’ in KEGG pathways (Figure 5A–C). Notably, genes related to photosystems I and II, the light harvesting complex and the cytochrome b6-f complex were mostly upregulated at an elevated CO_2_ concentration of 720 ppm, corresponding to *P. densiflora*’s photosynthesis adaptation response to elevated CO_2_. The expression levels of genes of the Calvin cycle and PPP, such as *RuBisCo* (*RBCS-1A* and *RBCS-1B*, EC:2.2.1.1), *Rubisco activase* (*RCA*) and *phosphoglycerate kinase 1* (*PGK1*, EC:2.7.2.3), were upregulated at an elevated CO_2_ concentration of 720 ppm (Figure 5D). Collectively, the expression levels of *RuBisCo* as well as its activator were found to increase at elevated CO_2_ concentration of 560 and 720 ppm as an adaptive measure to the increased photosynthesis. Meanwhile, the relative gene expression of some genes involved in the Calvin cycle and pentose phosphate pathways (PPP) showed two opposing trends at elevated CO_2_ concentrations of 560 and 720 ppm. In particular, *glyceraldehyde-3-phosphate dehydrogenase* (*GAPCP1*, EC:1.2.1.12) and *transketolase-1* (*TKL-1*, *TKL-2*, EC:2.2.1.1) genes were downregulated at the CO_2_ concentration of 560 ppm but upregulated at the CO_2_ concentration of 720 ppm (Figure 5D). The concentration of the indispensable photosynthetic pigment chlorophyll and its metabolic pathway were also regulated by CO_2_ levels. Here, we found increased expression of the *chlorophyll an oxygenase* (*CAO*) gene, an enzyme that catalyzes the conversion of chlorophyll(ide) a to chlorophyll b in elevated CO_2_ of 720 ppm (Figure 5E). Apart from the *CAO* gene, which mainly functions in the chlorophyll cycle, certain *chlorophyll biosynthesis* genes were also upregulated in pine leaves treated with an elevated CO_2_ concentration of 720 ppm (Figure 3E). Interestingly, *ferrochelatase-2* (*FC2*), expressed primarily in photosynthetic tissue, showed a downward trend at the elevated CO_2_ concentration of 560 ppm and increased expression at the elevated CO_2_ concentration of 720 ppm. FC2 catalyzes the formation of protoheme and is reported to be expressed in response to stress [24]. Hence, the opposing trend of *FC2* expression might be due to a differential adaptative response to increasing CO_2_ levels. Together, our results show that there is increased expression of genes involved in photosynthesis, carbon fixation, chloroplast synthesis and cycle at the CO_2_ concentration of 720 ppm.

### 2.5. Starch and Sucrose Metabolism

**Figure 6:**
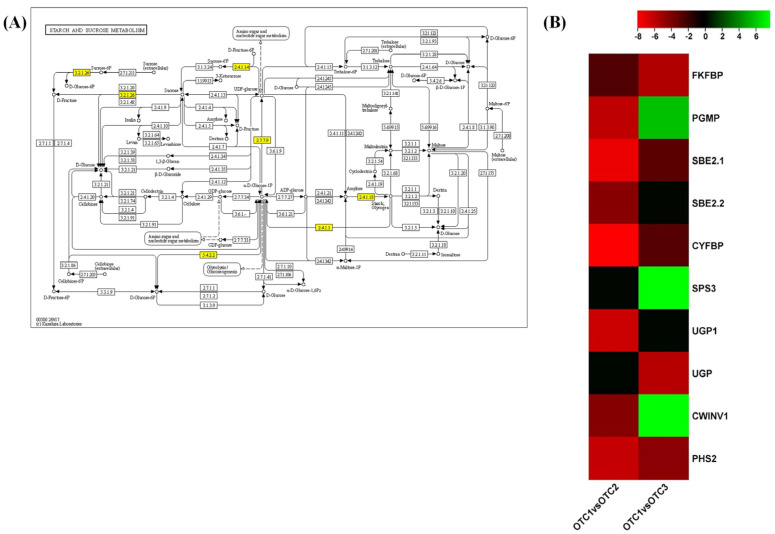
KEGG pathway enrichment of starch and sucrose metabolism. (**A**) KEGG pathway illustration showing DEGs involved in the starch and sucrose metabolic pathways. The yellow boxes indicate the DEGs in response to increased CO_2_. (**B**) Heatmap showing expression changes in the starch and sucrose pathways. The color bar shows the log2fold changes scale. Each species was treated with three different concentrations of CO_2_. OTC1 (control, ambient CO_2_ concentration of 400 ppm), OTC2 (elevated CO_2_ concentration of 560 ppm) and OTC3 (elevated CO_2_ concentration of 720 ppm).

Stimulated photosynthesis causes an increase in carbohydrate production under elevated CO_2_ concentrations [25]. However, in our study, we observed downward trends in starch- and sucrose-metabolizing genes. Ten genes of the starch and sucrose pathways were differentially regulated between ambient and elevated CO_2_ levels (Figure 6A). Markedly, key genes such as *6-phosphofructo-2-kinase* (*FKFBP*), *phosphoglucomutase* (*PGMP*, EC: 5.4.2.2) and *starch branching enzymes* (*SBE2.1*, *SBE 2.2*, EC: 2.4.1.18) of starch biosynthesis and *fructose-1,6-bisphosphatase* (*CYFBP*), *UTP glucose-1-phosphate uridylyltransferase 1* (*UGP*), *beta-fructofuranosidase* (*CWINV1*, EC: 3.2.1.26) and *α-glucan phosphorylase 2* (*PHS2*, EC: 2.4.1.1) of sucrose metabolism were downregulated at the elevated CO_2_ concentration of 560 ppm (Figure 6B). However, the genes *PGMP* and *CWINV1* shifted toward upregulation at the elevated CO_2_ concentration of 720 ppm. Similarly, *SBE*, *CYFBP* and *PHS2* showed increased expression compared with the elevated CO_2_ concentration of 560 ppm pine leaves but downregulated expression compared with the pine grown under ambient CO_2_ levels. Carbon partitioning between sucrose and starch was catalyzed by the crucial enzyme FKFBP, and its expression continuously declined progressively with increasing CO_2_ concentrations (Figure 6B). Another rate-limiting enzyme of sucrose synthesis as a photosynthetic product is *sucrose-phosphate synthase 3* (*SPS3*, EC:2.4.1.14), which was upregulated in pine leaves treated with an elevated CO_2_ concentration of 720 ppm, concluding a carbon portioning toward sucrose in an increased CO_2_ environment. UGP (EC:2.7.7.9) catalyzes the formation of UDP-glucose and pyrophosphate from glucose-1-phosphate and uridine triphosphate (UTP). The generated UDP glucose can be channeled to various carbohydrate synthesis pathways [16,26,27]. The expression of *UGP* was downregulated at both elevated CO_2_ levels.

### 2.6. Glycolysis/Gluconeogenesis

**Figure 7:**
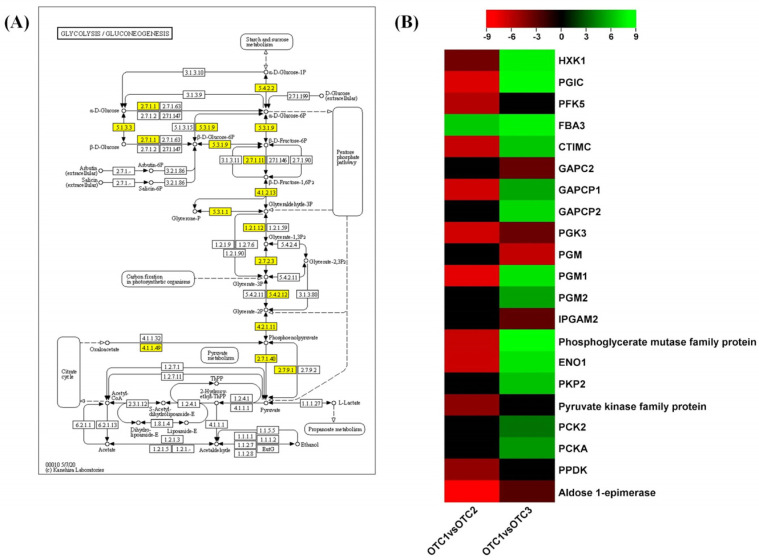
KEGG pathway enrichment of glycolysis and gluconeogenesis. (**A**) KEGG pathway illustration showing DEGs involved in glycolysis and gluconeogenesis. The yellow boxes indicate the DEGs in response to increased CO_2_. (**B**) Heatmap showing the differential expression of genes involved in glycolysis and gluconeogenesis. The color bar shows the log2fold changes scale. Each species was treated with three different concentrations of CO_2_. OTC1 (control, ambient CO_2_ concentration of 400 ppm), OTC2 (elevated CO_2_ concentration of 560 ppm) and OTC3 (elevated CO_2_ concentration of 720 ppm).

Glycolysis occurs both in plastids and cytosol in plants, and the differential expression of both plastidic and cytosolic enzymes was noted. To this end, *hexokinase* (*HXK1*, EC:2.7.1.1), *glucose-6-phosphate isomerase* (*PGIC*, EC:5.3.1.9), *triosephosphate isomerase* (*CTIMC*, EC:5.3.1.1), *glyceraldehyde-3-phosphate dehydrogenase* (*GAPC2*, EC:1.2.1.12), *phosphoglycerate kinase* (*PGK3*, EC:2.7.2.3) and pyruvate kinase family proteins are cytosolic glycolytic genes, while *6-phosphofructokinase 5* (*PFK5*, EC:2.7.1.11), *fructose-bisphosphate aldolase 3* (*FBA3*, EC:4.1.2.13), *GAPCP1*, *GAPCP2*, *phosphoglycerate mutase* (*PGM*, *PGM1*, *PGM2*, EC:5.4.2.11), *enolase* (*ENO1*, EC:4.2.1.11) and *plastidial pyruvate kinase 2* (*PKP2*, EC:2.7.1.40) are plastidial genes (Figure 7A). The expression levels of the observed glycolysis genes were decreased at the elevated CO_2_ concentration of 560 ppm compared with the ambient control. In contrast, these genes along with *PKP2* were upregulated in leaves at the elevated CO_2_ concentration of 720 ppm (Figure 7B). FBA3, which synthesizes D-glyceraldehyde 3-phosphate and dihydroxyacetone phosphate from fructose-1,6-phosphate, was upregulated in both elevated CO_2_ treatments (Figure 7B). This is the central step in the glycolysis/gluconeogenesis pathway, and the expression of *plastidic FBA3* was continually upregulated in elevated CO_2_ treatments. Phosphoenolpyruvate carboxykinase (PCK, EC:4.1.1.49) and pyruvate, phosphate dikinase 1 (PPDK, EC:2.7.9.1) are two important enzymes that direct two variable pathways of gluconeogenesis [28]. The expression of *PCK* was upregulated at the elevated CO_2_ concentration of 720 ppm, while *PPDK* was downregulated at the elevated CO_2_ concentration of 560 ppm.

### 2.7. H_2_O_2_ Stress-Related Signaling

**Figure 8:**
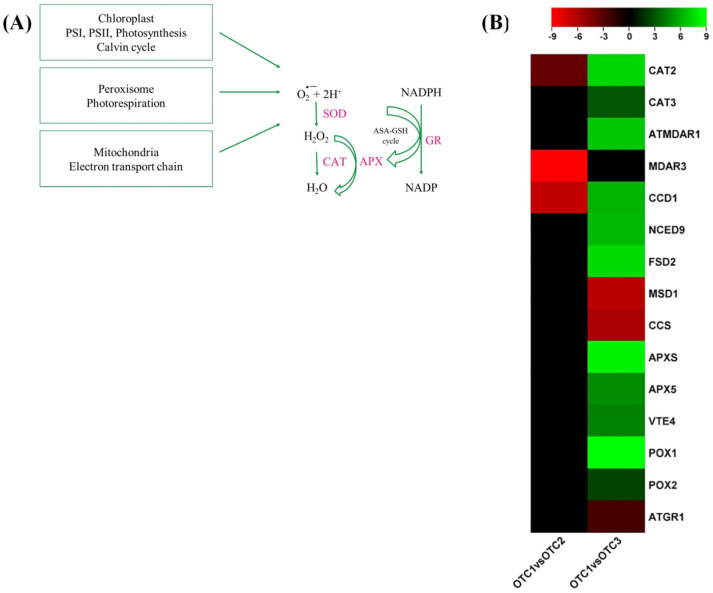
Enrichment of antioxidants in response to elevated CO_2_. (**A**) Antioxidant enzymes differentially regulated in the elevated CO_2_ levels. (**B**) Heatmap showing the differential expression patterns of antioxidants in response to increased CO_2_. The color of the bar shows the log2fold changes scale. Each species was treated with three different concentrations of CO_2_. OTC1 (control, ambient CO_2_ concentration of 400 ppm), OTC2 (elevated CO_2_ concentration of 560 ppm) and OTC3 (elevated CO_2_ concentration of 720 ppm). SOD, superoxide dismutase; CAT, catalase; GR, glutathione reductase; APX, ascorbate peroxidase; ASA-GSH cycle, ascorbate–glutathione cycle.

Photosynthesis and the electron transport chain are the major sources of physiological reactive oxygen species (ROS) generation, and environmental stresses have also been demonstrated to increase ROS production. Hydrogen peroxide (H_2_O_2_) is a potent free radical known to cause oxidative damage to cellular macromolecules. ROS scavenging is executed by enzymatic and nonenzymatic antioxidants. Four major antioxidant genes were differentially expressed compared to the ambient CO_2_ treatment (Figure 8A). The expression levels of 3 antioxidant genes, *catalase-2* (*CAT-2*), *monodehydroascorbate reductase 3* (*MDAR3*) and *carotenoid 9,10(9′,10′)-cleavage dioxygenase* (*CCD1*), were downregulated at an elevated CO_2_ concentration of 560 ppm (Figure 8B). Treatment at an elevated CO_2_ concentration of 720 ppm increased the gene expression levels of *catalase*, *MDAR*, *superoxide dismutase [Fe] 2* (*FSD2*), *L-ascorbate peroxidase* (*APXS*, *APX5*) and *tocopherol O-methyltransferase* (*VTE4*). Superoxide-related genes, *MSD1* and *CCS* and *glutathione reductase* (*ATGR1*) were downregulated at the elevated CO_2_ concentration of 720 ppm (Figure 8B). The increase in photosynthesis at elevated CO_2_ levels and the resultant increase in H_2_O_2_ might account for the increase in the stress-responsive antioxidant system observed in this study.

### 2.8. Validation of RNA-Seq Results by qPCR

**Figure 9:**
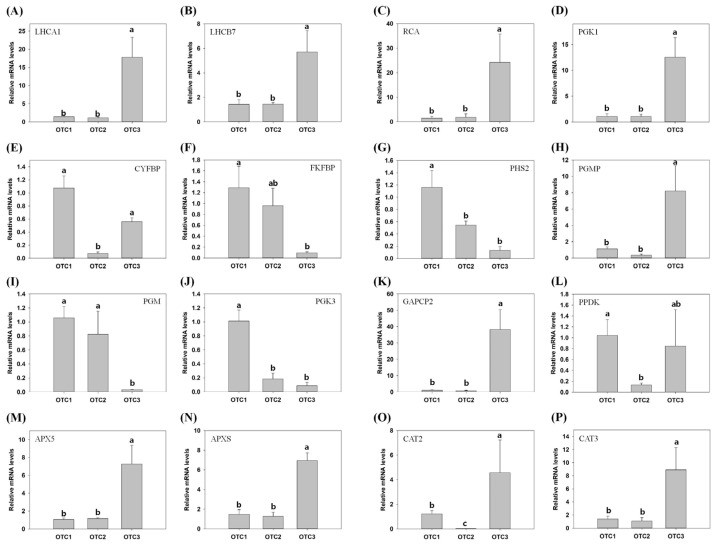
Validation of the differential expression of 16 genes by quantitative real-time PCR (qPCR). qPCR data were analyzed using the 2^−ΔΔCt^ method with the *TUB* and *U2AF* genes as internal controls. Three biological replicates were performed for each sample. Error bars show the mean standard error (*n* = 3). (**A**) *Chlorophyll a-b binding protein* (*LHCA1,* TRINITY_DN58729_c0_g1_i1). (**B**) *Chlorophyll a-b binding protein* 7 (*LHCB7,* TRINITY_DN78962_c0_g1_i2). (**C**) *Ribulose bisphosphate carboxylase/oxygenase activase* (*RCA,* TRINITY_DN87649_ c0_ g1_i1). (**D**) *Phosphoglycerate kinase* (*PGK1,* TRINITY_DN63836_c0_g1_i1). (**E**) *Cytosolic fructose-1,6-bisphosphatase* (*CYFBP,* TRINITY_DN35036 _c0_g1_i5). (**F**) *Fructose-2,6-bisphosphatase* (*FKFBP,* TRINITY_DN147783_ c0_g2_i1). (**G**) Cytosolic alpha-glucan phosphorylase 2 (*PHS2,* TRINITY _DN26074_c0_g1_i4). (**H**) *Chloroplastic phosphoglucomutase* (*PGMP,* TRINITY_DN64563_c1_g1_i5). (**I**) Phosphoglycerate mutase-like protein (*PGM,* TRINITY_DN64994_c0_g1_i2). (**J**) *Phosphoglycerate kinase* (*PGK3,* TRINITY_DN34875_c0_g6_i2). (**K**) *Chloroplastic glyceraldehyde-3-phosphate dehydrogenase 2* (*CAPCP2,* TRINITY_DN99376_c0_g1_i4). (**L**) *Chloroplastic phosphate dikinase 1* (*PPDK,* TRINITY_DN5478_c3_g1_i3). (**M**) *Peroxisomal L-ascorbate peroxidase 5* (*APX5,* TRINITY_DN60793_c0_g1_i1). (**N**) *Chloroplastic/mitochondrial L-ascorbate peroxidase S* (*APXS,* TRINITY _DN19267_c0_g1_i6). (**O**) *Catalase 2* (*CAT2,* TRINITY_DN169836_ c0_g1_i4). (**P**) *Catalase 3* (*CAT3,* TRINITY_DN278558_c0_g1_i1). Different lowercase letters indicate significant differences (ANOVA with Tukey’s HSD, *p* < 0.05).

We performed qPCR analysis for representative DEGs, including chlorophyll a-b binding protein (LHCA1), chlorophyll a-b binding protein 7 (LHCB7), ribulose bisphosphate carboxylase/oxygenase activase (RCA), phosphoglycerate kinase (PGK1), cytosolic fructose-1,6-bisphosphatase (CYFBP), fructose-2,6-bisphosphatase (FKFBP), cytosolic alpha-glucan phosphorylase 2 (PHS2), chloroplastic phosphoglucomutase (PGMP), phosphoglycerate mutase-like protein (PGM), phosphoglycerate kinase (PGK3), chloroplastic glyceraldehyde-3-phosphate dehydrogenase2 (CAPCP2), chloroplastic phosphate dikinase 1 (PPDK), peroxisomal L-ascorbate peroxidase 5 (APX5), chloroplastic/mitochondrial L-ascorbate peroxidase S (APXS), catalase 2 (CAT2) and catalase 3 (CAT3) (Figure 9 and Supplementary Table S2). To verify the differences in gene expression among OTC1, OTC2 and OTC3, 16 genes were selected for qPCR. In the qPCR assay, most of the genes showed similar expression patterns as were observed in the RNA-Seq data. We found that the photosynthetic pathway DEGs LHCA1, LHCB7, RCA and PGK1 were downregulated in OTC3 compared with OTC1. With the exception of the upregulated PGMP in OTC3, CYFBP was downregulated in OTC2, FKFBP was downregulated in OTC3 and PHS2 was downregulated in both OTC2 and OTC3 compared with OTC1. In the case of glycolysis and gluconeogenesis, PGM, PGK3 and PPDK were downregulated in OTC3, in both OTC2 and OTC3, and in OTC2 compared with OTC1, respectively. In addition, GAPCP2 was upregulated in OTC3. We analyzed the antioxidants in response to elevated CO_2_. Compared with OTC1, OTC3 showed upregulation of the DEGs APX5, APXS and CAT3, while CAT2 was downregulated in OTC2 and upregulated in OTC3.

## 3. Discussion

The physiological response of trees to increased CO_2_ is highly dependent on the species, developmental stage, growth period, additional nutrients and temperature [5,29,30]. We observed changes in *P. densiflora* grown in OTCs without any additional factor intervention. However, a temperature difference (up to 1.2–2 °C) higher than the outside was observed in OTC due to limited ventilation. The growth of pine trees at elevated CO_2_ concentrations of 560 ppm and 720 ppm showed increasing trends (Figure 1). In plants, increased primary carbon sources accelerate their metabolism and growth, especially when other growth sources are abundant [5,31]. Previous studies have reported that plants exhibit reduced growth due to alterations in primary metabolism after prolonged exposure to high CO_2_ concentrations and insufficient nitrogen [32]. In a recent report, the growth rate (height and diameter) of pine trees during CO_2_ elevation showed different growth rates in the early (1 year for CO_2_ elevation treatment) and mid-term (2–7 years for CO_2_ elevation treatment) [33]. In conclusion, a high concentration of CO_2_ treatment for 10 years in this study did not result in statistically significant differences in the height and diameter growth of pine trees.

We evaluated fifteen physiological traits. Of these, chlorophyll a, chlorophyll b and carotenoid contents are photosynthesis-related traits, five other traits are related to metabolites (total soluble sugar, glucose, fructose, sucrose and starch) and the remaining seven traits are related to stress tolerance (proline, H_2_O_2_, MDA, SOD, CAT, APX, anthocyanin). There have been previous reports that changes in the external environment may affect chlorophyll levels [34]. Our results confirmed that the levels of chlorophyll a, chlorophyll b and carotenoids showed significant changes (Figure 2). The changes in atmospheric CO_2_ concentration affect chlorophyll content in plants, which in turn is related to photosynthetic ability [35,36]. It has been reported that the rate of photosynthesis is reduced because of feedback of photosynthetic products and reduction in Rubisco content for long-term treatment, although photosynthetic rates of plants increase in the initial stage [37]. Referring to the above, our results showed that treatment with a high concentration of CO_2_ for 10 years resulted in long-term reactions. In particular, decreases in chlorophyll a at 720 ppm and chlorophyll b at both 560 ppm and 720 ppm, which are related to carbon fixation, are regulated by the feedback of accumulated starch and soluble sugar (Table 1 and Table 2) [36,38]. At elevated CO_2_ concentrations, the levels of chlorophyll a and chlorophyll b not only decreased the total chlorophyll content (chlorophyll a + b) but also changed the ratio of chlorophyll a/b. The ratio of chlorophyll a/b at both elevated CO_2_ concentrations increased compared with that under ambient conditions (400 ppm). Under various stress conditions, chlorophyll a and b decrease, chlorophyll b decreases more than chlorophyll a and the a/b ratio tends to increase [39]. As a result of the transcriptome analysis, chlorophyll synthesis-related genes were found to be hardly changed in OTC2, or only *FC2* decreased, whereas in OTC3, the expression levels of all of related genes were increased (Figure 5E). This is inferred to be related to the result of a significant decrease in the total chlorophyll content in OTC3. Unfortunately, no change in the expression of *Chl b reductase* (*CBR*) and *Mg-dechelatase* (*SGR*), a key gene for chlorophyll degradation, could be observed in our transcriptome analysis. Chlorophyll a constitutes various chlorophyll protein complexes in the photochemical system and carbon fixation system, and most chlorophyll b is used in the construction of light-harvesting chl-protein complexes in the photochemical system [36,38]. Therefore, it is presumed that the significant decrease in chlorophyll b may have lowered the activity of the photochemical system rather than the carbon fixation system. In a recent report, it was shown that in plants grown at high CO_2_ concentrations, the content of carotenoids changed [40]. In our results, the level of carotenoids increased at an elevated CO_2_ of 560 ppm (Figure 2). *CCD1* in transcriptome analysis was increased in OTC3 and decreased in OTC2. Increased expression of *CCD1* has a negative role in the accumulation of carotenoids, converting carotenoids to apocarotenoids (Table 1 and Figure 8B) [41]. It has been reported that photosynthetic genes are affected by the level of soluble sugar content in cells [30]. We found an increase in the soluble sugar at elevated CO_2_ concentrations, and these seemed to influence a decrease in the level of chlorophyll a at an elevated CO_2_ concentration of 720 ppm and the level of chlorophyll b at both elevated CO_2_ concentrations (Table 1 and Table 2). The increase in the concentration of atmospheric CO_2_ is likely to have significant effects on the photosynthesis, metabolism and development of plants [42,43]. The nitrogen content of the leaves can decrease, and photosynthetic products do not move to the sink organs and accumulate as starch, reducing the rate of photosynthesis [37]. This photosynthetic acclimation is also consistent with the significant increase in starch content at the elevated CO_2_ concentration of 560 ppm in our results (Table 2). Excessive carbohydrate accumulation due to high concentrations of CO_2_ is thought to have induced the overall degradation of genes involved in sucrose and starch synthesis (Table 2 and Figure 6). For instance, in OTC2, the expression levels of the starch branch synthesis-related genes *SBE2.1* and *SBE2.2* were decreased, but the starch content was increased, which is presumed to be the result of negative feedback due to excessive carbohydrate accumulation. Glucose and sucrose were reported to play the role of substrates for osmolytes and cellular respiration to maintain cell homeostasis in plants [44]. In contrast, fructose is not related to osmoprotection and is related to the synthesis of secondary metabolites [45]. Our results showed that the level of glucose increased at an elevated CO_2_ concentration of 720 ppm, and the level of sucrose increased at an elevated CO_2_ concentration of 560 ppm.

We measured the level of proline that increased at an elevated CO_2_ concentration of 560 ppm, which is supposed to improve drought tolerance by an osmotic pressure regulator (Figure 2B). It is known that high CO_2_ in the atmosphere has an advantage in drought stress tolerance of plants because it can keep the stomata small [46]. At the same time, the increases in antioxidants such as SOD, CAT, APX, carotenoids and anthocyanins, as well as the increases in glucose and sucrose acting as osmolalities, identified in this study may also affect the increase in drought tolerance under high CO_2_ concentration conditions. As reported previously, H_2_O_2_ is produced primarily by plant cells during photosynthesis and photorespiration and is affected by environmental stresses [47]. The level of H_2_O_2_ was significantly increased at an elevated CO_2_ concentration of 720 ppm in this experiment (Figure 2C). At the same time, the levels of SOD were significantly increased. Changes in the contents of H_2_O_2_ and SOD are correlated with each other [48]. SOD is known to catalyze the dismutation of superoxide (O_2_^−^) to H_2_O_2_ and is assumed to play a major role in providing defense against oxidative stress [49]. It is presumed that the accumulation of ROS as a result of the increases in photosynthesis- and glycolysis-related genes shown in the transcriptome analysis induced an increase in SOD and, as a result, an increase in H_2_O_2_ at an elevated CO_2_ concentration of 720 ppm. In addition, the antioxidants CAT and APX were increased to remove the increased H_2_O_2_ (Figure 2). CAT and APX are enzymatic H_2_O_2_ scavengers. APX is located in all cellular compartments where ROS are produced and regulates intracellular ROS steady-state levels. In contrast, CAT is located only in the peroxisome and has a lower H_2_O_2_ affinity than APX [48]. The related transcripts analyzed were *APXS*, *APX5*, *FSD2* and *MSD1* (Figure 8). *APXS* and *APX5* are APX synthesis-related genes and are sensitive to H_2_O_2_ concentration. Additionally, in this study, it was observed that, along with the increase in H_2_O_2_ in OTC3, the expression levels of APX synthesis-related genes and APX enzyme activity increased (Figure 2E and Figure 8B). Fe-SOD is sensitive to H_2_O_2_ concentration, and the *FSD2* gene was involved in this transcriptome analysis [50]. However, MSD1-associated Mn-SOD was not sensitive to the H_2_O_2_ concentration. From the results of the transcriptome analysis, we observed that *FSD2* increased in OTC3 and that *MSD1* decreased, which is also consistent with the results of this study, in which increases in H_2_O_2_ and SOD were observed in OTC3 (Figure 2C,D and Figure 8B). Anthocyanin is increased by abiotic stress and has been implicated in stress tolerance, such as ROS scavengers, photo protectants and stress signals [51]. The cause of the increase in anthocyanin content at an elevated CO_2_ concentration of 720 ppm is that carbon availability is improved or N content is decreased (Figure 2G) [32]. At an elevated CO_2_ concentration of 720 ppm, increases in the expression levels of antioxidant-related genes and enzyme activity were confirmed, but the MDA level did not show a significant change (Figure 2A). Therefore, these changes are thought to be due to an increase in photosynthesis-related factors rather than abiotic stress factors. As a result, in the stress-related response, the activity levels of antioxidant-related indicators such as SOD, CAT, APX, CAT and anthocyanin and the level of related genes were elevated at the CO_2_ concentration of 720 ppm, while only proline was elevated the CO_2_ concentration of 560 ppm.

In this study, it was revealed that the gene expression of pine trees grown at elevated CO_2_ showed different expression patterns depending on the CO_2_ concentration. However, some genes showed the same expression patterns at both elevated CO_2_ concentrations. In both OTC2 and OTC3, *FBA3* was upregulated and *PGK3*, *aldose 1-epimerase* (EC: 5.1.3.15), *FKFBP*, *SBE 2.1*, *CYFBP* and *PHS2* were all downregulated. It is expected that the expression levels of these genes will be continuously regulated by elevated CO_2_ concentrations.

## 4. Materials and Methods

### 4.1. Plant Materials and Growth Conditions

One-year-old pine (*P. densiflora*) seedlings were grown in pots containing appropriate soil moisture in the greenhouse and then transferred to open-top chambers (Figure 1A). Three plants were used in the experiments (Supplementary Figure S1). The experiment was conducted for 10 years at the National Institute of Forest Science in Suwon, Korea (37°15′04″ N, 136°57′59″ E), under natural environmental conditions [52]. Three CO_2_ concentrations were applied to the OTCs (decagon chamber, 10 m in diameter by 10 m in height); ambient (×1.0, ~400 ppm, OTC1), ×1.4 (~560 ppm, OTC2) and ×1.8 (~720 ppm, OTC3). Although the air temperature inside was 1.2–2.0 °C higher than that outside, the temperature differences among the OTCs were less than 0.2 °C. Pine leaves were harvested during the daytime in June 2019. Samples were stored at −80 °C until the experiment.

### 4.2. Measurement of the Chlorophyll Content

The chlorophyll content was determined following the method of Sibley et al. [9,53]. Fresh leaves, each containing 0.1 g of plant tissue, were taken in triplicate.

### 4.3. Extraction and Measurement of Soluble Sugar

Total soluble sugars were extracted from leaves with 80% ethanol and employing a modified method [9,54]. Fresh leaves, each containing 0.1 g of plant tissue, were taken in triplicate. The total soluble sugar content was measured at 620 nm by a Biospectrometer (Eppendorf, Hamburg, Germany) using glucose as the standard. The contents of soluble sugars are expressed as mg g^−1^ FW.

Glucose, fructose and sucrose were extracted from the leaves using the method in Lu and Sharkey [55]. Fresh leaves, each containing 0.1 g of plant tissue, were taken in triplicate. The sugar concentrations were measured as described Stitt et al., 1989 and a Biospectrometer [56] (Eppendorf, Hamburg, Germany). To analyze starch content, the resulting sediments from aqueous ethanol extractions were autoclaved for 3 h in distilled H_2_O and enzymatically digested to glucose according to the method described by Walters et al. [57]. α-amylase and amyloglucosidase from the Total Starch Kit were used to digest amylose and amylopectin into glucose. (Megazyme International Ireland Ltd.,Wichlow, Ireland, K-TSTA-100A). The sugar concentrations were determined enzymatically with a method described by Stitt et al., 1989 using a Biospectrometer [56] (Eppendorf, Hamburg, Germany).

### 4.4. Measurement of MDA, Proline, H_2_O_2_, SOD, CAT, APX and Anthocyanin

Proline was extracted from a sample of 0.5 g fresh leaves in 3% (*w*/*v*) aqueous sulfosalicylic acid and estimated using the ninhydrin reagent according to the method of Bates et al., 1973 [19]. The absorbance was read at a wavelength of 520 nm. The proline concentration was determined using a calibration curve and expressed as mmol proline g^−1^ FW. The experiments described below were performed in triplicate with approximately 0.1 g to 0.2 g of homogenized pine leaves. For measurement of MDA, pine leaves were extracted with 20% TCA (*w*/*v*) and 0.5% thiobarbituric acid (TBA) (*w*/*v*), followed by warming at 95 °C for 30 min. The mixture was placed on ice for 30 min and then centrifuged at 14,000× *g* for 10 min. The absorbance of the supernatant was read at 532 nm using a Biospectrometer (Eppendorf, Hamburg, Germany). The MDA content was derived according to the method of Heath and Packer, 1968 [58]. H_2_O_2_ was determined after reaction with 1 M KI and 100 mM K-phosphate buffer (pH 7.0). The reaction was incubated for 1 h in darkness, and the absorbance was measured at 390 nm. The amount of hydrogen peroxide was calculated using a standard curve prepared with known concentrations of H_2_O_2_ [59]. Superoxide dismutase (SOD) in leaves was determined using a superoxide dismutase (SOD) assay kit (Sigma-Aldrich Crip, St. Louis, MO, USA), and catalase (CAT) activity was analyzed using a catalase (CAT) assay kit (cat. DG-CAT400, Dogen, Seoul, Korea). A plant ascorbate peroxidase (APX) kit (MBS2602897, MyBioSource, San Diego, CA, USA) was used to determine APX activity in homogenized leaf samples. All procedures were performed according to kit protocols. The optical density was measured immediately at 450 nm, 560 nm and 450 nm after preparation using an automated plate reader (SpectraMax M2, Molecular Devices, San Jose, CA, USA). Anthocyanin was extracted by the method described by Winter and Huber, 2000 [27]. The amount of anthocyanin was determined as an absorption difference, A_530_–A_657_ (SpectraMax M2, Molecular Devices, San Jose, CA, USA).

### 4.5. RNA Isolation, Library Preparation, RNA Sequencing Analysis and qRT-PCR Analysis

Total RNA was isolated from the pine leaves of three biological replicates for each CO_2_ treatment using an RNeasy plant mini kit (Qiagen). Approximately 2 μg of RNA from each tissue was used to construct cDNA libraries for sequencing according to the NEBNext2 Ultra RNA library Prep kit preparation protocol. Briefly, the polyadenylated RNA molecules were isolated using poly-T oligo-attached magnetic beads, followed by enzymatic RNA fragmentation, cDNA synthesis, ligation of bar-coded adapters and PCR amplification to create cDNA libraries. The generated libraries were sequenced using a Novaseq 6000 PE150 platform with 150 paired-end sequences. The obtained raw reads were quality checked using FastQC before any subsequent analysis and assembly. Then, adapter sequences were removed by Trimmomatic software (v0.0.14) [60]. Furthermore, trimmed and quality-checked reads were assembled leveraging the Trinity RNA-Seq de novo transcriptome assembly platform. The assembled SAM files were then converted to BAM format by SAMtools [61], and the Feature Counts tool was used to estimate the uniquely mapped gene counts [62].

For real-time quantitative RT-PCR (qPCR) analysis, DNase-treated total RNA was converted to cDNA using cDNA EcoDry^TM^ Premix (TaKaRa, Shiga, Japan). Directed qPCR was conducted using a CFX96 Touch Real-Time PCR Detection System (BIO-RAD, Hercules, CA, USA) with IQ^TM^ SYBR Green Supermix (BIO-RAD, CA, USA) according to the manufacturers’ instructions. The gene-specific primers used for qPCR are listed in Supplementary Table S1. The conditions for the reaction were as follows: 95 °C for 30 s, 38 cycles of 95 °C for 5 s and 60 °C for 34 s. Three independent biological replicates and three technical replicates for each biological replicate were run. The 2^−ΔΔCt^ method was used to analyze relative transcript abundance (Livak and Schmittgen, 2001) [63]. The expression levels of *TUB* and *U2AF* were used for the normalization of quantitative real-time PCR results [64].

### 4.6. Differentially Expressed Gene (DEG) Analysis

The generated gene count files from each condition were used for differential expression gene analysis using the DEseq2 package in the R analysis environment [65]. Furthermore, Fisher’s exact test and likelihood ratio test methods were implemented to perform differential expression analysis, following a binomial distribution. In addition, the Benjamini-Hochberg method was used for multiple hypothesis testing of the *p* values of genes. Genes with |log2 (fold change)| >  2 and *p* value  < 0.05 were classified as differentially expressed genes.

### 4.7. Functional Annotation of DEGs

The Omicsbox (Blast2GO) program (Biobam, Valencia, Spain, https://www.biobam.com/omicsbox/ (accessed on 10 October 2021)) was used to perform functional annotation analysis. In detail, we performed a homology search based upon the BLASTx program for the *P. densiflora* gene sequences against the Arabidopsis protein database (NCBI Arabidopsis protein sequences, https://ftp.ncbi.nlm.nih.gov/genomes/all/GCF/000/001/735/GCF_000001735.4_TAIR10.1/GCF_000001735.4_TAIR10.1_protein.faa.gz (accessed on 10 October 2021)) using a cutoff E-value of 10^−5^, and the maximum number of allowed hits was fixed to 10 per query. Only the alignment results with the smallest E-value and query coverage were considered to select the best top hits. Furthermore, the DEGs were GO annotated and pathway enriched using gene set enrichment analysis in WebGestalt [22]. The heatmap was constructed with TBtools using the log2 fold change of DEGs [66].

### 4.8. Statistical Analysis

Analyses were conducted using one-way ANOVA with multiple comparisons using Tukey’s HSD. *p* values < 0.05 were considered significant. Values are presented as the means with SD.

## 5. Conclusions

In this study, we investigated the changes in the physiology, biochemistry and transcriptome of pine trees grown over a long period of 10 years at elevated CO_2_ concentrations under both mild and severe conditions. Overall, elevated CO_2_ concentrations of 560 ppm and 720 ppm showed different overall patterns. As a result of physiological analysis, at an elevated CO_2_ concentration of 560 ppm, chlorophyll content decreased, carotenoids increased and both chlorophyll a and b significantly decreased. The total unstructured carbohydrate content increased overall, especially for sucrose and starch at an elevated CO_2_ concentration of 560 ppm and glucose at an elevated CO_2_ concentration of 720 ppm. Notably, the H_2_O_2_ content, antioxidant enzymatic activity and related gene expression levels were significantly increased at an elevated CO_2_ concentration of 720 ppm. Transcriptome analysis of photosynthesis, sucrose and starch synthesis, the glucose/glycolysis pathway and antioxidant-related genes showed different patterns at elevated CO_2_ concentrations of 560 ppm and 720 ppm. The expression levels of genes related to photosynthesis, the electron transport chain and carbon fixation were elevated only at an elevated CO_2_ concentration of 720 ppm. The expression levels of genes in the sucrose and starch synthesis pathway were decreased at an elevated CO_2_ concentration of 560 ppm and partially increased at an elevated CO_2_ concentration of 720 ppm, which is thought to be the result of suppressive feedback due to excessive carbohydrate accumulation at an elevated CO_2_ concentration of 560 ppm when inferred along with the content analysis results. In addition, most genes related to the glycolysis/gluconeogenesis pathway showed very opposite patterns at elevated CO_2_ concentrations of 560 ppm and 720 ppm. The expression levels of related genes were increased at an elevated CO_2_ concentration of 720 ppm and decreased at an elevated CO_2_ concentration of 560 ppm. This DEG analysis suggests that the carbon fixation action and the ATP production induction in pine trees react differently depending on the CO_2_ concentration. The results of this study showed an acclimatizing response as commonly reported in long-term treated pines at the mildly elevated CO_2_ concentration of 560 ppm. However, in the case of a higher concentration of CO_2_ of 720 ppm, photosynthesis, carbon fixation and carbohydrate production were still promoted at the genetic level, despite a decrease in chlorophyll content and an increase in carbohydrate content. The contradictory results of the reaction of pine trees according to the long-term CO_2_ concentration show that the research direction should be set differently depending on the expected increase in atmospheric CO_2_ concentration in the future, and these findings are expected to be helpful in suggesting the research direction.

## Figures and Tables

**Table 1:** Effects of elevated CO_2_ on the photosynthetic pigments in *P. densiflora*.

Treatment	mg/g FW ^(w)^				Chl a/b ^(w)^	Chl/Car ^(w)^
	Chl a	Chl b	Total Chl	Carotenoids		
OTC1 ^(z)^	0.99 ± 0.13 ^a^	0.37 ± 0.08 ^a^	1.35 ± 0.20 ^a^	0.34 ± 0.04 ^b^	2.72 ± 0.26 ^b^	3.92 ± 0.20 ^a^
OTC2 ^(y)^	1.05 ± 0.07 ^a^	0.20 ± 0.01 ^b^	1.35 ± 0.08 ^a^	0.44 ± 0.03 ^a^	5.17 ± 0.13 ^a^	3.06 ± 0.01 ^b^
OTC3 ^(x)^	0.80 ± 0.07 ^b^	0.15 ± 0.02 ^b^	1.02 ± 0.09 ^b^	0.34 ± 0.03 ^b^	5.49 ± 0.39 ^a^	3.04 ± 0.04 ^b^

^(z)^ OTC1, open-top chamber 1 (control, ambient CO_2_ concentration of 400 ppm); ^(y)^ OTC2, open-top chamber 2 (elevated CO_2_ concentration of 560 ppm); ^(x)^ OTC3, open-top chamber 3 (elevated CO_2_ concentration of 720 ppm); ^(w)^ The values are the means ± SDs (*n* = 3). Different lowercase letters indicate significant differences (ANOVA with Tukey’s HSD, *p* < 0.05).

**Table 2:** Concentrations of glucose, fructose, sucrose and total soluble sugars on a fresh weight basis in pine leaves.

Treatment	mg/g FW ^(z)^				
	Glucose	Fructose	Sucrose	Starch	Total Soluble Sugar
OTC1 ^(z)^	9.53 ± 0.78 ^b^	5.80 ± 0.93	5.97 ± 1.42 ^b^	2.71 ± 1.31 ^b^	50.90 ± 4.18 ^b^
OTC2 ^(y)^	11.25 ± 3.68 ^ab^	6.32 ± 1.13	7.81 ± 0.70 ^a^	4.20 ± 0.79 ^a^	62.75 ± 5.31 ^a^
OTC3 ^(x)^	11.69 ± 1.37 ^a^	6.79 ± 1.95	7.83 ± 2.99 ^ab^	3.73 ± 1.53 ^ab^	57.06 ± 1.00 ^a^

^(z)^ OTC1, open-top chamber 1 (control, ambient CO_2_ concentration of 400 ppm); ^(y)^ OTC2, open-top chamber 2 (elevated CO_2_ concentration of 560 ppm); ^(x)^ OTC3, open-top chamber 3 (elevated CO_2_ concentration of 720 ppm); ^(w)^ The values are the means ± SDs (*n* = 3). Different lowercase letters indicate significant differences (ANOVA with Tukey’s HSD, *p* < 0.05).

**Table 3:** Summary of RNA sequencing and de novo assembly.

Pre-Processing and Mapping			
Features	OTC1 ^(z)^	OTC2 ^(y)^	OTC3 ^(x)^
Raw reads (2 × 150 bp)	29,696,002	31,143,622	29,022,614
Data in GB	5.46	5.71	5.35
Filtered reads (bp)	29,409,471	30,821,126	28,728,623
Q30 (%)	95.31	95.19	95.24
GC (%)	45.97	46.12	45.69
Number of mapped reads (%)	70.72	70.31	68.72
Trinity de novo assembly			
Total assembled bases	80,433,621		
Number of transcripts	74,868		
Average transcript length (bp)	1074		

^(z)^ OTC1, Open-top chamber 1 (control, ambient CO_2_ of 400 ppm); ^(y)^ OTC2, Open-top chamber 2 (elevated CO_2_ of 560 ppm); ^(x)^ OTC3, Open-top chamber 3 (elevated CO_2_ of 720 ppm).

## Data Availability

The data presented in this study are available on the NCBI SRA database (PRJNA907482, accessed on 2 December 2022).

## Supplementary Materials

The following supporting information can be downloaded at: https://www.mdpi.com/article/10.3390/plants11243530/s1, Table S1: Primers of qPCR in *Pinus Densiflora*. Table S2: Genes/transcripts information of qPCR in *Pinus Densiflora*. Figure S1: The photographs of nine *Pinus Densiflora* in Open-Top Chambers (OTCs).
