# Supplementary material for: Elevated CO2 Alters the Physiological and Transcriptome Responses of Pinus densiflora to Long-Term CO2 Exposure
Source: Plants (Basel). 2022 Dec 15;11(24):3530. doi: 10.3390/plants11243530 (PMC9781706; doi:10.3390/plants11243530)
Supplement: Supplementary file 1 [file plants-11-03530-s001.zip › Supplementary figure 1.pdf]

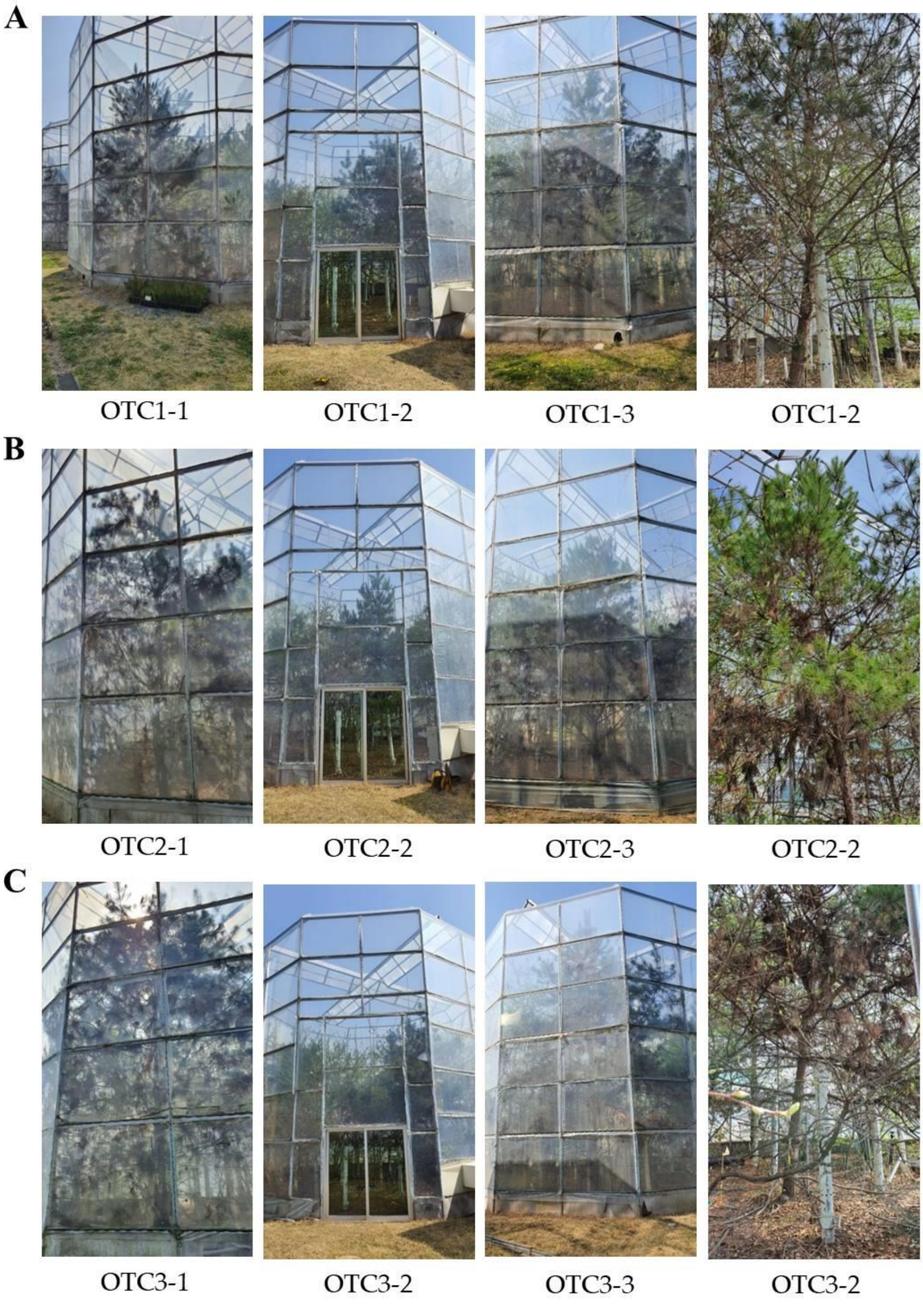

Supplementary Figure S1. The photographs of nine *Pinus Densiflora* in Open-Top Chambers (OTCs). (A) Three pine trees in OTC1 facility. There are OTC1-1, OTC1-2, OTC1-3 from the left in the picture. (B) Three pine trees in OTC2 facility. There are OTC2-1, OTC2-2, OTC2-3 from the left in the picture. (C) Three pine trees in OTC3 facility. There are OTC3-1, OTC3-2, OTC3-3 from the left in the picture.
