# Supplementary material for: Elevated CO2 Alters the Physiological and Transcriptome Responses of Pinus densiflora to Long-Term CO2 Exposure
Source: Plants (Basel). 2022 Dec 15;11(24):3530. doi: 10.3390/plants11243530 (PMC9781706; doi:10.3390/plants11243530)
Supplement: Supplementary file 1 [file plants-11-03530-s001.zip › Supplementary table 1.pdf]

Supplementary Table S1. Primers of qPCR in *Pinus Densiflora*

Gene	Forward primer (5′→3′)	Reverse primer (5′→3′)
GAPCP2	CAGCCTGGACAACAAGATCA	CTGCTTCCCACTCTCCACAT
PGK3	ACAGTCTGCAACCTGTCGTG	GCTTTGACCTTCTTGCCATC
PGM	CCGTCGGGTTATAAGGGATT	ATTCTCGGGACTCCTTGGTT
PPDK	GCCGTCTACGACCAACTGAT	AGTGTCTCGGTGTCGTCGAT
CAT2	CAAGACTACCGACCCACTCG	GTGTTGTTGGCAACCATGTC
CAT3	TACCACGAGTGCAAGGTCAG	TCTCGCCATGCTTGTAGTTG
APXS	TCACCTCAAGCAACTGCAAC	CTCGTACTCGTCTGCGACTG
APX5	CTTGAGCCCCAGAAGAACTG	ACCAGGTGTGAAGGGAACAG
LHCB7	ATCTCTGTCTGCCAGGTGCT	GCCGTTTTTGATTTCCTTGA
LHCA1	CAGGTTCAGGGAGAGTGAGC	TGCAATGAGGGTGTTGATGT
RCA	GTCCCCCTGATTCTGGGTAT	ACATCCTGCCCTTCTTGATG
PGK1	ACTGCCAAGTGGAAAGCAGT	TCGGTGATCTTCTGGCTCTT
FKFBP	CAGTCCTCGTGGTTTCCAAT	TGTGAGACCGTCGCAGTTAC
PGMP	TGGAAGTTCTTCTGCGGTCT	AAGTCGTGCTGGATGCTCTT
CYFBP	TGGTGGTATCTTCGCCTACC	TCTGCACCTCGTCGTAACTG
PHS2	GAACTGCGATTGAAGCAACA	CTCAAGGCCTTCCAAGTCAG
U2af	TCGGGAGGTTGGGTCTACAT	ACCAGTCCTTCAGTCCCCTT
β-TUB	GTCGTGAATCATGGCATGGC	GCCTCACTATCGGTTTCCCA
