# Supplementary material for: Elevated CO2 Alters the Physiological and Transcriptome Responses of Pinus densiflora to Long-Term CO2 Exposure
Source: Plants (Basel). 2022 Dec 15;11(24):3530. doi: 10.3390/plants11243530 (PMC9781706; doi:10.3390/plants11243530)
Supplement: Supplementary file 1 [file plants-11-03530-s001.zip › supplementray table 2.pdf]

Supplementary Table S2. Genes/transcripts information of qPCR in *Pinus Densiflora*

Pine tree transcripts name	Arabidopsis Gene symbol	Arabidopsis Description
TRINITY_DN58729_c0_g1_i1	*LHCA1*	Chlorophyll a-b binding protein
TRINITY_DN78962_c0_g1_i2	*LHCB7*	Chlorophyll a-b binding protein 7
TRINITY_DN87649_ c0_ g1_i1	*RCA*	Ribulose bisphosphate carboxylase/oxygenase activase
TRINITY_DN63836_c0_g1_i1	*PGK1*	Phosphoglycerate kinase
TRINITY_DN35036 _c0_g1_i5	*CYFBP,*	Cytosolic fructose-1,6-bisphosphatase
TRINITY_DN147783_ c0_g2_i1	*FKFBP*	Fructose-2,6-bisphosphatase
TRINITY _DN26074_c0_g1_i4	PHS2	Cytosolic alpha-glucan phosphorylase 2
TRINITY_DN64563_c1_g1_i5	*PGMP*	Chloroplastic phosphoglucomutase
TRINITY_DN64994_c0_g1_i2	*PGM*	Phosphoglycerate mutase-like protein
TRINITY_DN34875_c0_g6_i2	*PGK3*	Phosphoglycerate kinase
TRINITY_DN99376_c0_g1_i4	*CAPCP2*	Chloroplastic glyceraldehyde-3-phosphate dehydrogenase 2
TRINITY_DN5478_c3_g1_i3	*PPDK*	Chloroplastic phosphate dikinase 1
TRINITY_DN60793_c0_g1_i1	*APX5*	Peroxisomal L-ascorbate peroxidase 5
TRINITY _DN19267_c0_g1_i6	*APXS*	Chloroplastic/mitochondrial L-ascorbate peroxidase S
TRINITY_DN169836_ c0_g1_i4	*CAT2*	Catalase 2
TRINITY_DN278558_c0_g1_i1	*CAT3*	Catalase 3
